# Energy-efficient information transfer at thalamocortical synapses

**DOI:** 10.1371/journal.pcbi.1007226

**Published:** 2019-08-05

**Authors:** Julia Jade Harris, Elisabeth Engl, David Attwell, Renaud Blaise Jolivet

**Affiliations:** 1 Department of Neuroscience, Physiology & Pharmacology, University College London, London, United Kingdom; 2 Department of Life Sciences, Imperial College London, South Kensington Campus, London, United Kingdom and Francis Crick Institute, London, United Kingdom; 3 Département de Physique Nucléaire et Corpusculaire (DPNC), University of Geneva, Geneva, Switzerland and CERN, Geneva, Switzerland; University of Hertfordshire, United Kingdom

## Abstract

We have previously shown that the physiological size of postsynaptic currents maximises energy efficiency rather than information transfer across the retinothalamic relay synapse. Here, we investigate information transmission and postsynaptic energy use at the next synapse along the visual pathway: from relay neurons in the thalamus to spiny stellate cells in layer 4 of the primary visual cortex (L4SS). Using both multicompartment Hodgkin-Huxley-type simulations and electrophysiological recordings in rodent brain slices, we find that increasing or decreasing the postsynaptic conductance of the set of thalamocortical inputs to one L4SS cell decreases the energy efficiency of information transmission from a single thalamocortical input. This result is obtained in the presence of random background input to the L4SS cell from excitatory and inhibitory corticocortical connections, which were simulated (both excitatory and inhibitory) or injected experimentally using dynamic-clamp (excitatory only). Thus, energy efficiency is not a unique property of strong relay synapses: even at the relatively weak thalamocortical synapse, each of which contributes minimally to the output firing of the L4SS cell, evolutionarily-selected postsynaptic properties appear to maximise the information transmitted per energy used.

## Author summary

Compared to other organs, the brain consumes a vast amount of energy for its size. Most of this energy is used to power the electrical and chemical processes that support neural computation. As the energy supply to the brain is limited, it follows that this computation should be energetically efficient. Previously, we showed that this is indeed the case for transmission of information between cells at synapses. Synapses transferring information from the retina to the brain do not maximise information transmission—some information is lost and does not reach the visual cortex. Instead, these synapses maximise the information transmitted per energy used. Here, we demonstrate that this principle of energetic efficiency also holds at the next synapse in the visual pathway, the thalamocortical synapse. This synapse is weaker and competes with hundreds of other inputs to influence the output firing of the next cell. Using detailed simulations of cortical neurons, and electrophysiological recordings in rodent brain slices, we found that this relatively weak synapse also does not maximise information transmission. Instead, it maximises the amount of information transmitted per energy used. This suggests that energy efficiency at synapses could be a common design principle in the brain.

## Introduction

Information transmission in the brain is energetically expensive [1–7], yet has to satisfy demands of speed and signal-to-noise reliability [8, 9]. In order to balance these competing demands, the brain may tend towards a design which prioritises energy efficiency at the expense of computational power. For instance, theoretical analysis has previously shown that the low mean firing rate of neurons [10] and the surprisingly low release probability of cortical synapses [4, 11] can be explained if axons and presynaptic terminals operate to maximise the information transmitted per energy used.

Experimentally, we have shown previously that the strong retinothalamic synapse, relaying information from the retina to the thalamus, also maximises energetic efficiency when transferring information [12]. At that synapse, the evolutionarily selected properties are not set to transmit the maximum amount of information possible—more information would be transmitted if larger excitatory postsynaptic currents (EPSCs) were evoked by presynaptic action potentials. However, EPSCs that are larger or smaller than physiological EPSCs decrease the information transmitted per energy used. The physiological EPSC size therefore maximises energy efficiency of information transfer rather than information transfer across the retinothalamic synapse. Crucially however, it is unclear whether energy efficiency at excitatory synapses is a special property of strong relay synapses, or a more general principle also governing synaptic inputs that contribute more weakly to determining the output of the postsynaptic cell.

To address this question, we investigated information transmission and EPSC energy cost at the next synapse along the visual pathway, from relay neurons in the thalamus to spiny stellate (SS) cells in layer 4 (L4) of the primary visual cortex (V1). This relatively weak thalamocortical synapse operates in the presence of many other synaptic inputs from the thalamus and from the cortex. Using a multicompartment Hodgkin-Huxley-type model of L4SS cells, we simulated random synaptic bombardment from thalamic and cortical synapses, while quantifying the energetic cost of information transmission across the synapses impinging on the cortical cell from a single thalamic axon. We assessed the energetic cost incurred by these postsynaptic cells in V1 by converting the ion flows across the membrane resulting from EPSCs or from action potentials into the corresponding amount of adenosine triphosphate (ATP) molecules necessary to pump these ions back out [1, 4, 12]. At the same time, we evaluated information transfer from an axon of interest to the output spike train of these cells using transfer entropy [13, 14]. Similar to what we observed at retinothalamic synapses, our simulations suggested that the energetic efficiency of information transmission was maximal at the physiologically observed level of thalamocortical synapse conductance.

Then, we tested this result experimentally on real L4SS cells patch-clamped in rat brain slices, evoking thalamocortical input while using dynamic-clamp to inject the random background synaptic conductance derived from our simulations. We found that increasing or decreasing the conductance at thalamocortical synapses decreased the energetic efficiency of information transmission from one, experimentally-stimulated, thalamic axon.

Thus, both simulations and experiments suggest that, like at the retinothalamic synapse [12], thalamocortical postsynaptic properties are evolutionarily set to be energy efficient.

## Materials & methods

### Ethics statement

P28 Sprague Dawley rats were killed by cervical dislocation following sedation with isoflurane. Animal procedures were carried out in accordance with the guidelines of the UK Animals (Scientific Procedures) Act 1986 and subsequent amendments.

### Mathematical model of spiny stellate cells

The mathematical model of L4SS cells was adapted from an earlier model by Lavzin and colleagues [15] available at ModelDB (https://senselab.med.yale.edu/ModelDB/showmodel.cshtml?model=146565) [16]. The multicompartment simulations were conducted using the NEURON 7.3 simulation platform (http://neuron.yale.edu/) and will be available from our GitHub page (https://github.com/JolivetLab).

The cell was subdivided into 360 compartments, with a maximum length of 21 μm (14.6 μm on average). Following Lavzin et al. [15], the soma area was 757 μm^2^, the total dendritic area was 11,885 μm^2^, the resting membrane potential was -70 mV, the membrane resistance was 16,000 Ω·cm^2^, the axial resistivity was 100 Ω·cm and the membrane capacitance in all compartments was set to 1.5 μF/μm^2^ to account for the presence of spines.

The model included Hodgkin-Huxley-type voltage-gated channels. Fast sodium channels (reversal potential = 70 mV), and delayed rectifier and slow non-inactivating potassium channels (reversal potential = -77 mV), were distributed with a higher density at the soma (gNa = 300 mS/cm^2^, gKdr = 30 mS/cm^2^, gKs = 100 mS/cm^2^) than in the dendritic tree (gNa = 3 mS/cm^2^, gKdr = 1 mS/cm^2^, gKs = 1 mS/cm^2^). L-type voltage-gated calcium channels were distributed evenly across the cell (gCa = 0.03 pS/μm^2^). Calcium diffusion within and between compartments was modelled as described in Carnevale and Hines [17] with a diffusion coefficient of 0.6 μm^2^/ms, and an intracellular calcium buffer at 3 μM concentration with a dissociation constant of K_d_ = 1 μM. Calcium pumping across the surface membrane was modelled again as described in [17] with a plasma membrane calcium pump density of 10^−13^ mol/cm^2^. The pump is modelled with a two-step reaction. The first step describes binding of intracellular calcium to the pump, with a binding rate constant of k_1_ = 1 mM^-1^ms^-1^ and an unbinding rate constant of k_2_ = 0.005 ms^-1^ (giving an apparent dissociation constant of 5 μM). The second step describes loss of the calcium from the pump to the extracellular solution, with an unbinding rate constant of k_3_ = 1 ms^-1^ and a rebinding rate constant of k_4_ = 0.005 mM^-1^ms^-1^ (giving an apparent dissociation constant of 200 mM). See Chapter 9 of Carnevale and Hines [17] for further details.

**Fig 1:**
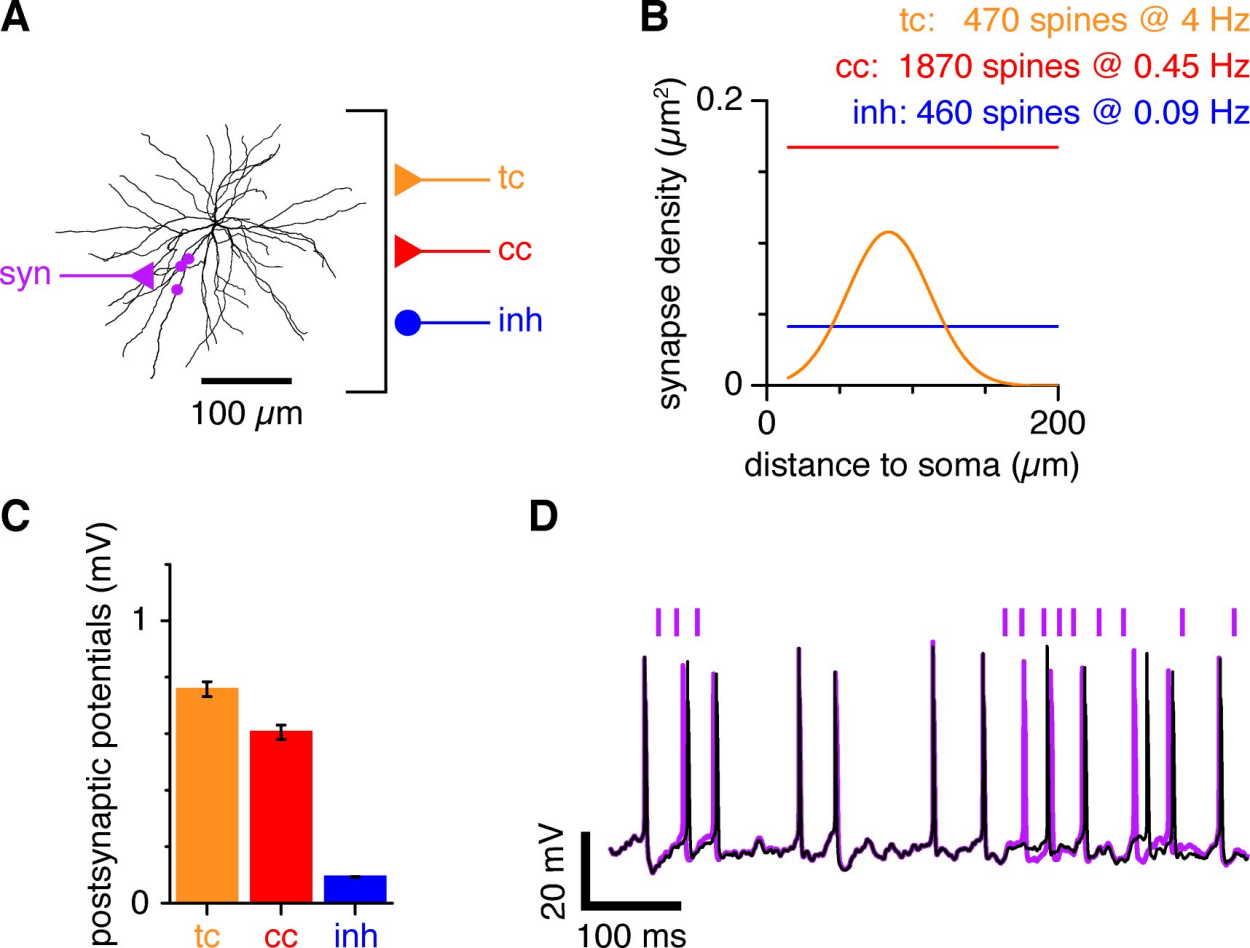
Hodgkin-Huxley-type multicompartment model of a layer 4 spiny stellate cell (L4SS). (A) To study energetic efficiency at thalamocortical synapses, we adapted a model of a L4SS cell [15]. The modelled cell receives excitatory (cc; red) and inhibitory (inh; blue) corticortical input, and thalamocortical input (tc; orange). Information transfer and energy consumption were investigated for input synapses from a single projecting thalamic neuron (syn; light violet). To model this additional projecting neuron, three synaptic contacts were added in close proximity along one dendritic branch. (B) Corticocortical input density (red and blue) for each type of synapse per μm^2^ of dendrite, as a function of distance from the soma over all dendrites. Thalamocortical input density followed a Gaussian probability distribution with respect to distance from the soma (orange; Gaussian with a mean of 83.6 μm and a standard deviation of 28.3 μm, transformed to show density per μm^2^; [19]). (C) The individual synaptic conductance values were the same for all synapses in one class. Excitatory conductances were adjusted to the values stated in Materials & Methods to generate EPSPs at the soma of ~0.6–0.8 mV depending on synapse location (plotted as mean ± standard deviation; ranges 0.29–2.65 mV for thalamocortical input and 0.25–2.32 mV for excitatory corticocortical input). Inhibitory conductances were set to produce IPSPs at the soma between 0.1 and 269 μV (mean 93 ± 31 μV), depending on synapse location. (D) Input from the projecting thalamic neuron of interest affects the output of the cell. Comparison between output generated by the background activity alone (black line; tc, cc and inh active) and when the projecting thalamic neuron of interest (syn) is activated on top of the background activity (light violet; timing of extra inputs marked as vertical bars above trace). The extra input is sufficient to alter the timing of output action potentials and, on occasion, to generate additional action potentials.

To simulate cortical background input to the modelled cell, one lumped excitatory and one lumped inhibitory synapse (each representing a number of synapses) were placed on each compartment of the model (Fig 1A). The frequency of activation of each lumped synapse was adjusted by the size of the compartment area so as to model monosynaptic inputs onto 1870 excitatory and 460 inhibitory spines homogeneously distributed throughout the dendritic tree at densities of 0.167/μm^2^ and 0.041/μm^2^ respectively, which were randomly activated at 0.45 Hz and 0.09 Hz respectively (Fig 1B). Frequencies for the corticocortical background input were slightly adjusted from Waters and Helmchen [18], so that after adding thalamocortical synapses (see below), the modelled cell’s output frequency was ~4 Hz, the mean cortical firing rate *in vivo* [1]. Excitatory conductances were modelled with a time course of the form exp(-t/τ_fall_)—exp(-t/τ_rise_) with time constants τ_rise_ = 0.3 ms and τ_fall_ = 1.7 ms, and with the reversal potential at 0 mV. Inhibitory synapses were modelled with the same function with time constants τ_rise_ = 1 ms and τ_fall_ = 10 ms, and with the reversal potential at -75 mV. The conductance of these synapses was held constant at g_cc_ = 0.0008 μS and g_inh_ = 0.0005 μS. To test for the influence of inhibition, some simulations were run without inhibitory synapses.

To simulate thalamocortical background input to the modelled cell, one excitatory synapse was placed on each compartment and the frequency of activation of each one of those synapses was adjusted according to the distance of that compartment to the soma (Fig 1B). This is equivalent in the NEURON simulation environment to modelling 470 spines distributed as a Gaussian with respect to their position in the dendritic tree [19] and randomly activated at 4 Hz (the average output frequency we measured in LGN cells [12]), but is less computationally intensive. Thalamocortical synapses were modelled with a conductance time course of the form exp(-t/τ_fall_)—exp(-t/τ_rise_) with τ_rise_ = 0.3 ms and τ_fall_ = 1.7 ms, and with the reversal potential at 0 mV. Their conductance was initially set at g_tc_ = 0.001 μS.

To evaluate the contribution of an individual thalamic cell projecting onto the modelled cell, three synapses were added, clustered onto the same dendritic branch, approximately 78 μm away from the soma [19] and typically spaced by 18 μm (see Fig 1A and 1B for details; located on compartments a3_122, a3_121 and a3_12 at positions 0.5, 0.2 and 0.9 respectively, see file l4sscell.hoc in https://senselab.med.yale.edu/ModelDB/showmodel.cshtml?model=146565 for details). These synapses were modelled using the same parameters as for other thalamocortical synapses. In particular, their conductance was initially set at g_syn_ = 0.001 μS. This is to approximate in the model three synaptic sites, which together produce an EPSC of a similar magnitude to that observed in experiments (3_*_(conductance of 10^-9^S)_*_(driving force of -70mV) ~ 210pA; see below). These synapses were synchronously activated using the same input spike train as was used for the experiments described below in the presence of both thalamocortical, and excitatory and inhibitory corticocortical background noise. To determine the optimal conductance value for all thalamocortical synapses, we modulated both g_syn_ and g_tc_ by the same gain factor while maintaining g_cc_ and g_inh_ constant. None of the synapses exhibited plasticity, facilitation, depression or failures. Note however that these synapses typically express only mild depression (see below).

To test for the influence of the exact location of these three additional thalamocortical synapses on our results, we repeated all the simulations with a second set of such synapses (located on compartments a5_1111, a5_11111 and a5_11111 at positions 0.9, 0.15 and 0.1 respectively, see file l4sscell.hoc in https://senselab.med.yale.edu/ModelDB/showmodel.cshtml?model=146565 for details). These additional simulations revealed no qualitative differences from what had been obtained with the first set. The location of the first set of synapses is plotted in Fig 1A (syn).

Additionally, for experiments described below, we generated two synthetic “noise” conductance trains, one corresponding to the total excitatory corticocortical background input and one corresponding to the total thalamocortical background input. To generate these trains, simulations were run as described above with two exceptions. First, inhibitory input was ignored. Second, all synapses were relocated to the soma and their conductances were summed.

### Calculating synaptic energy use

**Fig 2:**
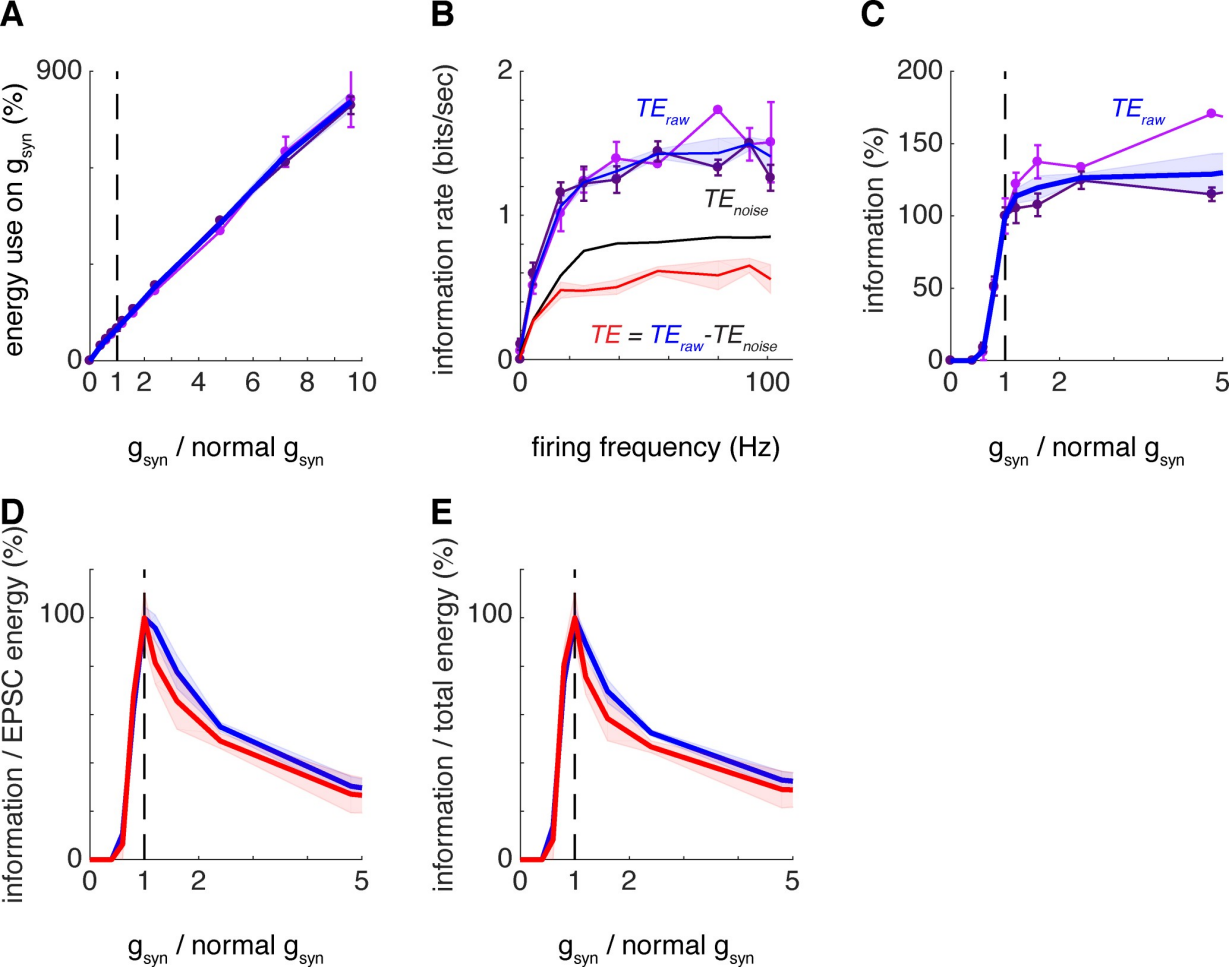
Postsynaptic conductance magnitude maximising information transferred per energy used in a model of spiny stellate cells. (A) Energy use on reversing postsynaptic ion influx for the synaptic contacts of interest as a function of g_syn_ multiplier (in all simulations the same multiplier is applied to g_tc_, while g_cc_ and g_inh_ remain constant). In panels A to C, two datasets are presented (pink and violet; error bars stand for the standard deviation) which resulted from placement of the synaptic contacts of interest on different dendritic branches (pink: 53.6, 70.9 and 88.0 μm away from the soma; violet: 67.5, 80.3 and 106.9 μm away from the soma). The position of the synaptic contacts had no qualitative effect on the results. The initial information transfer for the second set of contacts was *TE*_*raw*_ = 1.1 ± 0.1 bits/sec (as compared to 1.0 ± 0.1 bits/sec for the first set; *TE* = 0.47 ± 0.06 bits/sec is only calculated as an average over both datasets; mean ± s.e.m.). Additionally, in panels A to E, the average over both datasets is plotted (blue line; shaded areas stand for the standard error of the mean throughout). (B) Dependence of transfer entropy (between the specific input considered and the L4SS cell output) on mean output firing frequency evoked by input trains with different g_syn+tc_ multiplier values. Transfer entropy rates (*TE*_*raw*_) increase sigmoidally with the output firing frequency and plateau after approximately 20 Hz. As in panel A, the pink and violet traces stand for two different placements of the synaptic contacts of interest on different dendritic branches, while the blue line stands for the average over both datasets. Additionally, we plot *TE*_*noise*_ (black; mean ± s,e.m.) and the effective transfer entropy *TE = TE*_*raw*_—*TE*_*noise*_ (red; mean ± s.e.m.). See text and Materials & Methods for further details about the definition and meaning of *TE*_*raw*_, *TE*_*noise*_ and *TE*. (C) Dependence of the transfer entropy (*TE*_*raw*_) (“information”) on g_syn+tc_ multiplier plateaus as the multiplier is increased at a value slightly higher than its physiological value of 1. (D) Transfer entropy divided by energy use on reversing the ion influx at thalamocortical synapses shows a maximum at g_syn+tc_ multiplier value = 1. Both *TE*_*raw*_, the average over both datasets (blue), and *TE* (red) show maxima for metabolic efficiency of information transfer at 1. (E) Same as in D but when including the energetic cost of output action potentials in the calculation.

**Fig 3:**
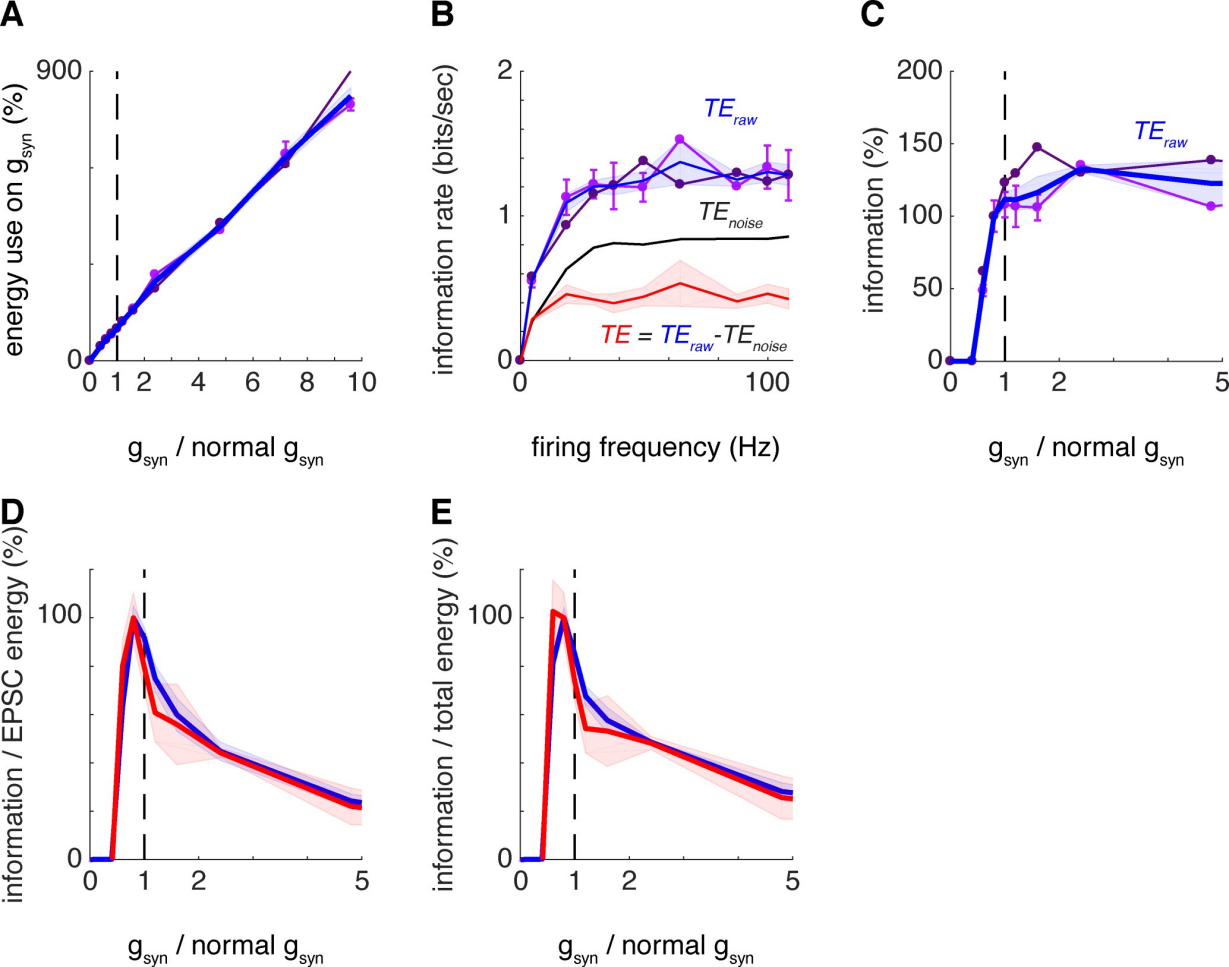
Inhibition modulates the position of peak energetic efficiency of information transfer. Same as in Fig 2 when omitting inhibition (setting g_inh_ to 0; see Materials & Methods). (A) Energy use on reversing postsynaptic ion influx for the synaptic contacts of interest as a function of g_syn_ multiplier. In panels A to C, two datasets omitting inhibition are presented (pink and violet; error bars stand for the standard deviation) which resulted from placement of the synaptic contacts of interest on the same different dendritic branches than in Fig 2. Like for Fig 2, the position of the synaptic contacts had no qualitative effect on the results. Additionally, in panels A to E, the average over both datasets is plotted (blue line; shaded areas stand for the standard error of the mean throughout). (B) Dependence of transfer entropy (between the specific input considered and the L4SS cell output) on mean output firing frequency evoked by input trains with different g_syn+tc_ multiplier values. Transfer entropy rates (*TE*_*raw*_) increase sigmoidally with the output firing frequency and plateau after approximately 20 Hz. Like in Fig 2, the blue line stands for the average over both datasets. Additionally, we plot *TE*_*noise*_ (black; mean ± s,e.m.) and the effective transfer entropy *TE = TE*_*raw*_—*TE*_*noise*_ (red; mean ± s.e.m.). (C) Dependence of the transfer entropy (*TE*_*raw*_) on g_syn+tc_ multiplier plateaus with the multiplier slightly higher than its physiological value of 1. Removal of inhibition slightly shifts the transition from 0 to maximal information transmission towards lower gain factors (compare with Fig 2C). (D) As a consequence, transfer entropy divided by energy use on reversing the ion influx at thalamocortical synapses shows a maximum at g_syn+tc_ multiplier < 1. Both *TE*_*raw*_, the average over both datasets (blue), and *TE* (red) show maxima for metabolic efficiency of information transfer at gain slightly < 1. (E) Same as in D but when including the energetic cost of output action potentials in the calculation. All curves peak at gain slightly < 1.

To calculate the metabolic cost incurred by the modelled cell (Figs 2 and 3), the total synaptic current generated by g_syn_, which is the largest signalling cost [1, 4], was integrated over the three synaptic locations and converted to the corresponding ATP consumption per unit time [1, 12]. The energetic cost of other synapses and output action potentials was calculated in the same way. For the experiments described below, the ATP used to reverse the postsynaptic ion flux was calculated for voltage-clamp and dynamic-clamp recording modes as described in [12].

### Transfer entropy

Using information theory in neuroscience has a long tradition [20]. In particular, it is common to use *mutual information* to quantify the flow of information from one neuron to another, or from a stimulus set to a recorded neuron [4, 12, 21, 22]. Numerous methods have been devised to measure mutual information in various contexts, and to correct for its inherent biases (see [23] for a review). However, mutual information is by construction a symmetric measure and thus does not strictly measure information being transferred from a sender to a receiver. Rather, when measuring mutual information between two random variables, one captures how much information can be inferred about one of those processes when observing the other one. Mutual information also suffers from significant estimation biases when the dataset is limited, a recurrent problem in experimental contexts like the one we will deal with here.

More recently, a measure analogous to mutual information called *transfer entropy*, which seeks to capture the unidirectional flow of information from one variable to another, was introduced [13], and has been increasingly applied to neuronal spike data (for comprehensive reviews of the application of transfer entropy in neuroscience, see [24–26]). Transfer entropy is non-symmetric. Let us define two processes *I* and *J* with joint probability *p*_*IJ*_*(i*,*j)*. The transfer entropy from *J* to *I* is defined by:

$${TE}_{J\to I}=\sum p(i_{n+1},i_{n}^{\left(k\right)},j_{n+1-u}^{\left(l\right)})\log\frac{p(i_{n+1}|i_{n}^{\left(k\right)},j_{n+1-u}^{\left(l\right)})}{p(i_{n+1}|i_{n}^{\left(k\right)})}$$ (1)

where $i_{n}^{\left(k\right)}=(i_{n},\ldots ,i_{n-k+1})$ is a shorthand notation for words of length *k*, and where *n* denotes the n^th^ time bin and *u* an optional frame shift (see below) [13]. The sum runs over all possible combinations of *i*_*n*_, $i_{n}^{\left(k\right)}$ and $j_{n}^{\left(l\right)}$. For words of length 1, in simple cases where information flow is strictly unidirectional and when consecutive bins are both independent and conditionally independent given the source value, it is possible to show analytically that transfer entropy is equivalent to mutual information. Simulations also suggest that transfer entropy is largely superior to mutual information in that its estimate converges much faster when increasing the size of the dataset when analysing spike trains (Conrad and Jolivet, *in preparation*). Transfer entropy is used extensively in fields outside neuroscience and in systems neuroscience but is used relatively little in cellular neuroscience. While we decided to use here transfer entropy instead of mutual information because of the technical reasons highlighted above, essentially the same results were obtained when we used mutual information. Similarly, there is no reason to think that we would have found different results in ref. [12] had we used transfer entropy instead of mutual information.

Here, we binarized the 125 second input and output spike trains in 3 ms time bins (approximately the refractory period of a neuron) and measured the transfer entropy from the input axon to the output spike train using the package published by Ito and colleagues (https://code.google.com/archive/p/transfer-entropy-toolbox/) [14]. The results of this procedure were divided by the time bin (3 ms) to get information rates in bits/sec [27].

The package by Ito and colleagues allows consideration of a temporal frame shift (*u*) between the sender and the receiver. We systematically analysed the effect of this parameter on the results and observed that peak information transfer always occurs in the same time bin in our simulations, i.e. that transfer entropy is maximal without a frame shift between the input and output sequences when using a 3 ms temporal resolution (S2 Fig). Using shorter bins yielded similar results (see S3 Fig). Nevertheless, in all subsequent analysis, we allowed for temporal frame shifts of up to 10 time bins (30 ms). Changing this value had no significant impact on the results.

Changing the word length however does have an effect on the measure of transfer entropy. (We talk here about word length in the singular, as we systematically considered the same word length for both the input and output sequences, thus *k = l*. See refs. [26] and [28] for best practice in choosing word lengths). Specifically, increasing the value of *k* increases the measured transfer entropy, because of bias increase (this is well documented [26–28]). With a finite dataset, random coincidences can lead to mis-estimating probabilities, which will add up in the final calculation of the transfer entropy product (see Eq 6 in [14]).

In order to remediate that problem, one solution is to subtract from our raw estimates of *TE*, the value of *TE*_*noise*_, calculated between a random permutation of the input sequence and the actual output sequence. We carried out a systematic analysis of the effect of varying *k* on *TE—TE*_*noise*_ and observed no significant changes in the main results of this paper when k ≤ 10. We thus carried out all of our analyses with *k = l = 10* (30 ms). All values reported in the results section are of *TE* defined as:

$$TE={TE}_{raw}-{TE}_{noise}$$ (2)

with *TE*_*raw*_ calculated following Eq (1) above and *TE*_*noise*_ calculated following Eq (1) above but with randomly permuting the input sequence (randomizing words instead of individual time bins produces almost identical results, see S1 Fig). In practice, for each condition, we generated multiple simulations with different seeds initialising the random number generator, averaging about 6 individual simulations for each condition. For each one of those, we calculated *TE*_*raw*_, 30 realisations of *TE*_*noise*_ over which we took an average, and *TE*. The values of *TE*_*noise*_ and *TE* reported in the Results section are the average over all these individual simulations.

### Analysis of energetic efficiency

For all conditions (simulations, and experiments with real stimulation and all dynamic-clamp gains described below), the information rate was divided by the rate of energy consumption on reversing the ion flux generating EPSCs and action potentials to get a measure of efficiency in bits/(ATP consumed). All data were analysed using custom-written MATLAB scripts (The Mathworks Inc.).

### Experimental design

In the second part of the present study, we sought to replicate our simulation results in *in vitro* experiments using rat brain slices. The first part of each experiment was performed in whole-cell voltage-clamp mode, in order to calculate the energy cost per EPSC. L4SS cells were patch-clamped and thalamic axons were stimulated with a previously recorded thalamic relay neuron response train to retinal input (see below). The second part of the experiment was performed by injecting the same stimulation pattern in current-clamp in order to measure information transmission across the synapse. From the voltage-clamp recording, a conductance trace was then generated and injected into the cell with dynamic-clamp. The conductance trace, to which noise was added to simulate the physiological background of additional synapses, was scaled up or down to measure information transmission at conductances above or below the physiological value. Energetic efficiency for each scale factor (gain) was calculated by dividing the transfer entropy rate by the rate of energy consumption [4, 12]. This allowed us to assess whether, as in the LGN [12], energetic efficiency at the thalamocortical synapse was maximised at the physiological gain.

### Slice preparation

P28 Sprague Dawley rats were killed by cervical dislocation following sedation with isoflurane. The brain was rapidly removed and immersed in ice-cold, slicing solution containing (in mM): 87 NaCl, 25 NaHCO_3_, 7 MgCl_2_, 2.5 KCl, 1.25 NaH_2_PO_4_, 0.5 CaCl_2_, 25 glucose, 75 sucrose, 1 kynurenic acid, saturated with 95% O_2_/5% CO_2_ (modified from ref. [29]).

The hemispheres were separated along the midline and an off-coronal cut (20° from vertical, heading anteriorly while cutting towards the dorsal surface, between the cerebrum and cerebellum) was performed on each hemisphere to create an angled surface, which was glued onto the stage of the vibratome. Off-coronal 225 μm slices containing the thalamus and cortex including V1 were then made.

Slices were placed in a storage chamber containing continuously oxygenated slicing solution at 35°C, which was allowed to come to room temperature naturally. During the experiment, slices were continuously perfused with artificial cerebrospinal fluid (aCSF) containing (in mM): 124 NaCl, 26 NaHCO_3_, 10 glucose, 2.5 KCl, 2 CaCl_2_, 1 NaH_2_PO_4_, 1 MgCl_2_, 0.005 Gabazine (to block disynaptic inhibition during stimulation). The aCSF was heated to 35°C and constantly bubbled with 95% O_2_/5% CO_2_.

### Electrophysiology

**Fig 4:**
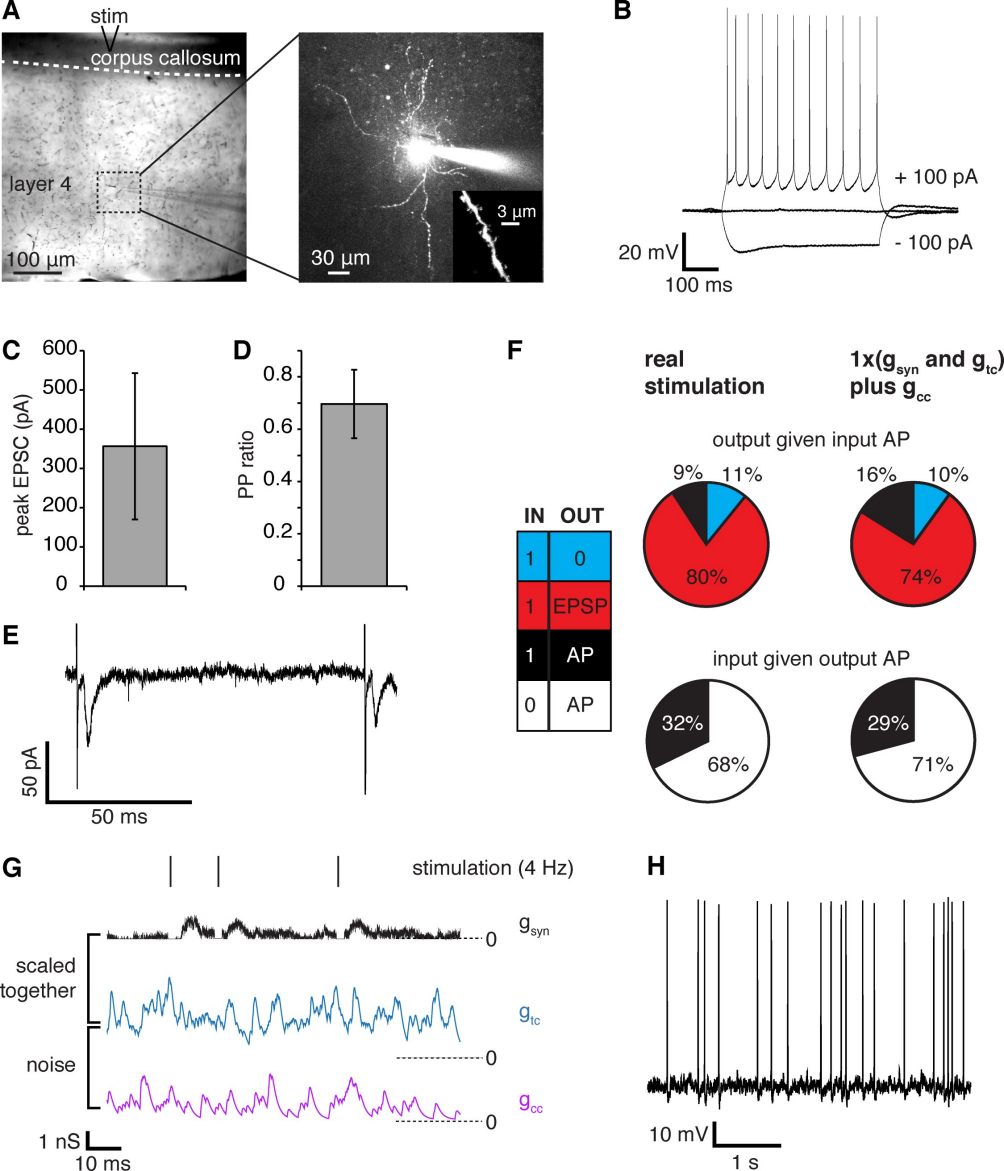
Characterisation of the thalamocortical synapse. (A) Specimen dye-filled spiny stellate (SS) neuron: its position in the slice (left, ‘stim’ shows stimulator position for stimulation of thalamic axons), non-pyramidal dendrite morphology (right) and visible spines (inset). (B) Typical response of L4SS neurons to injected current. (C) Mean amplitude of the first excitatory postsynaptic current (EPSC), n = 6 cells. (D) Paired pulse ratio (2^nd^/1^st^ EPSC) for 100 ms separation between pulses, n = 6 cells. (E) Typically, a mild paired pulse depression was observed with minimal stimulation (the vertical lines are stimulus artefacts 100 ms apart). (F) Observed input-output combinations (18ms search window, n = 6 cells) for presynaptic stimulation (left) and dynamic-clamp when noise is added (right). The possible combinations are given by the colour table (far left): input–no output (blue), input–output EPSP (red), input–output action potential (black), no input–output action potential (white). (G) L4SS neuron response in dynamic-clamp to injection of thalamocortical and corticocortical noise conductances, which were pre-scaled to achieve a firing frequency of ~4 Hz. (H) Specimen voltage response to injected conductances using dynamic-clamp. The synaptic conductance train recorded in response to presynaptic stimulation (g_syn_) was added to the pre-scaled thalamocortical (g_tc_, 20% of input synapses) and corticocortical (g_cc_, 80% of input synapses) noise conductance trains. For dynamic-clamp injection, g_syn_ and g_tc_ were multiplied by the same gain factor (1 for this recording), while g_cc_ remained constant.

Whole-cell recordings from cortical L4SS cells were obtained using 2–3 MΩ borosilicate glass electrodes filled with internal solution containing (in mM): 130 K-gluconate, 10 EGTA, 10 HEPES, 4 NaCl, 4 MgATP, 1 CaCl_2_, 0.5 Na_2_GTP, 0.4 K_2_-Lucifer yellow. Spiny stellate cells in layer 4 of cortical area V1 were identified according to their location, morphology, and electrophysiological characteristics (Fig 4A and 4B). In contrast to the less-numerous pyramidal neurons also found in L4, the round or ellipsoidal spiny stellate cells do not have a prominent apical dendrite or dendrites reaching across several layers, but have a broader dendritic tree confined to L4 [30, 31] with a high density of spines, which could be seen once a cell was dye-filled (Fig 4A). L4SS cells were recorded at their resting potential of approximately -70mV, at which they respond to current injection with sustained, regular spiking (Fig 4B; [32]).

Online corrections were made for the junction potential of -14 mV for the gluconate-based internal solution used (e.g. neurons were held at an apparent potential of -56 mV to achieve a true potential of -70 mV). Recordings were made with an Axopatch 200B amplifier, filtered at 5 kHz and sampled at 20 kHz. Data were acquired using custom-made MATLAB software, kindly provided by Ho Ko and Tom Mrsic-Flogel (UCL).

The first part of the experiment was performed in voltage clamp. Upon seal formation, pipette capacitance was compensated. Once in whole-cell mode, the series resistance was compensated by up to 70% (after which the mean series resistance was 6.4 ± 1.0 MΩ). The second part of the experiment was performed in current clamp, using the I-CLAMP FAST mode (which was stable with the 2–3 MΩ pipettes used). In current clamp mode, series resistance compensation was set to 100%.

Thalamic axons in the subcortical white matter were stimulated extracellularly with a borosilicate glass electrode (gently broken to achieve a tip diameter of ~10–15 μm) containing aCSF. In voltage clamp, stimulation strength was gradually increased to achieve the smallest reliable EPSC (defined as an EPSC that, when it occurred, did not vary in size in response to a pulse delivered every 3 s). This stimulus intensity was then maintained throughout the experiment. The average minimal EPSC size was 356 ± 186 pA (Fig 4C) and paired thalamic stimulation elicited the mild EPSC depression characteristic of this synapse (PPR = 0.70 ± 0.13, Fig 4D and 4E; [31, 33, 34]).

### Stimulation pattern

We stimulated thalamic axons making synapses onto whole-cell patch-clamped L4SS cells of V1 in rat brain slices (Fig 4A). The spike train used for stimulation was recorded from a typical LGN relay neuron in response to retinal ganglion cell (RGC) input [12], which in turn was recorded from RGCs in response to natural movies [35]. The spike train was 25 seconds long, with an average frequency of 4.3 Hz. The stimulation procedure followed that of our previous experiments in the LGN [12]: After a 5 second input train to habituate the synapse (the data from this train were discarded), the 25 second spike train was repeatedly played five times, resulting in 125 seconds of stimulation in total. This procedure was followed once in voltage-clamp, once in current-clamp, and several times in dynamic-clamp with various conductance gains (see below).

### Dynamic-clamp

A 125 second long conductance train (g_syn_) was derived from the 125 second postsynaptic current recording obtained from each spiny stellate cell in voltage clamp. First, we removed the stimulation artefacts by setting the current value for the duration of the artefact to the current value immediately preceding the artefact. The resulting current trace (I_syn_) was converted to a synaptic conductance trace (g_syn_) via:

$$g_{syn}\left(t\right)=I_{syn}\left(t\right)/(V_{m}-V_{rev})$$ (3)

where V_m_ is the membrane potential of the cell (the holding potential, -70 mV), and V_rev_ is the reversal potential of the synapse (0 mV, the reversal potential for glutamatergic ionotropic receptors).

In addition to the synaptic conductance trace, we mimicked the effect of this synapse operating in the presence of hundreds of other synaptic inputs. We generated two synthetic “noise” conductance trains, designed to have the characteristics of (1) thalamocortical (TC) inputs (4 Hz input to 470 spines: g_tc_) and (2) corticocortical (CC) inputs (0.45 Hz input to 1870 spines: g_cc_). These two trains were the result of simulations carried out before the experiment (see above the section ‘Mathematical model of spiny stellate cells’), and were the same for each cell. In subsequent dynamic-clamp experiments, the amplitude of the TC conductance noise was scaled with the conductance of the synapse being studied (g_syn_), since we assume that all the TC synapses will have their conductances set to the same optimal value. In contrast, the corticocortical noise was not scaled.

The baseline amplitude of the noise conductance was set individually for each cell by combining both trains (g_tc_+g_cc_) and scaling them up or down (mean scaling factor 0.4 ± 0.1) until a firing frequency of approximately 4 Hz [1] was triggered upon injection into the L4SS cell (“pre-scaling”, Fig 4G). The pre-scaled thalamic noise train (g_tc_) and the thalamic single-input train recorded in that cell (g_syn_) were then summed and scaled together (by a factor of 0.1, 0.3, 0.5, 0.75, 1, 1.5, and 3). In contrast to the LGN relay synapse [12], for which only the synaptic conductance was scaled up, in this experiment the combined synaptic and noise conductances could not be scaled above 3 times the physiological value without inducing oscillations or a depolarising block, resulting in a lack of action potential firing.

The pre-scaled corticocortical noise train (g_cc_) was then added to each of these scaled trains, to create 7 differently scaled composite conductance trains for each cell (Fig 4G). These trains were injected into the cell using dynamic-clamp (SM-1, Cambridge Conductance [36]), which injects a time-varying current I_inj_(t), at time t, calculated from g_syn_(t) and the instantaneous value of the cell membrane potential:

$$I_{inj}\left(t\right)=g_{syn}\left(t\right)\cdot (V_{m}\left(t\right)–V_{rev})$$ (4)

Because of the liquid junction potential, the V_m_ received by the SM-1 was 14 mV more positive than the real membrane potential. We therefore set V_rev_ on the SM-1 to 14 mV (rather than 0 mV), to account for this in the online calculation of I_inj_. In this calculation, all of the synaptic current was assumed to scale linearly with membrane potential. The voltage response of the postsynaptic cell was simultaneously recorded.

After injecting g_syn+tc_ /(normal g_syn+tc_)_*_1, the order of scaled conductances was randomized. This meant that not every cell experienced every scale factor, as it was not usually possible to maintain whole-cell recordings for long enough to carry out every conductance injection, after all initial steps were completed (i.e. minimal stimulation protocol, paired pulse ratio characterisation, voltage clamp response to real stimulation, current clamp response to real stimulation, noise pre-scaling to achieve 4 Hz firing rate, and finally various dynamic clamp conductance injections). As such, g_syn+tc_ /(normal g_syn+tc_)_*_1 was applied to all 6 cells, _*_0.3 to 3 cells, _*_0.5 to 4 cells, _*_0.75 to 5 cells, _*_1.5 to 5 cells, and _*_3 to 4 cells. The mean and SEM were calculated separately across these n numbers for each condition.

### Data analysis

**Fig 5:**
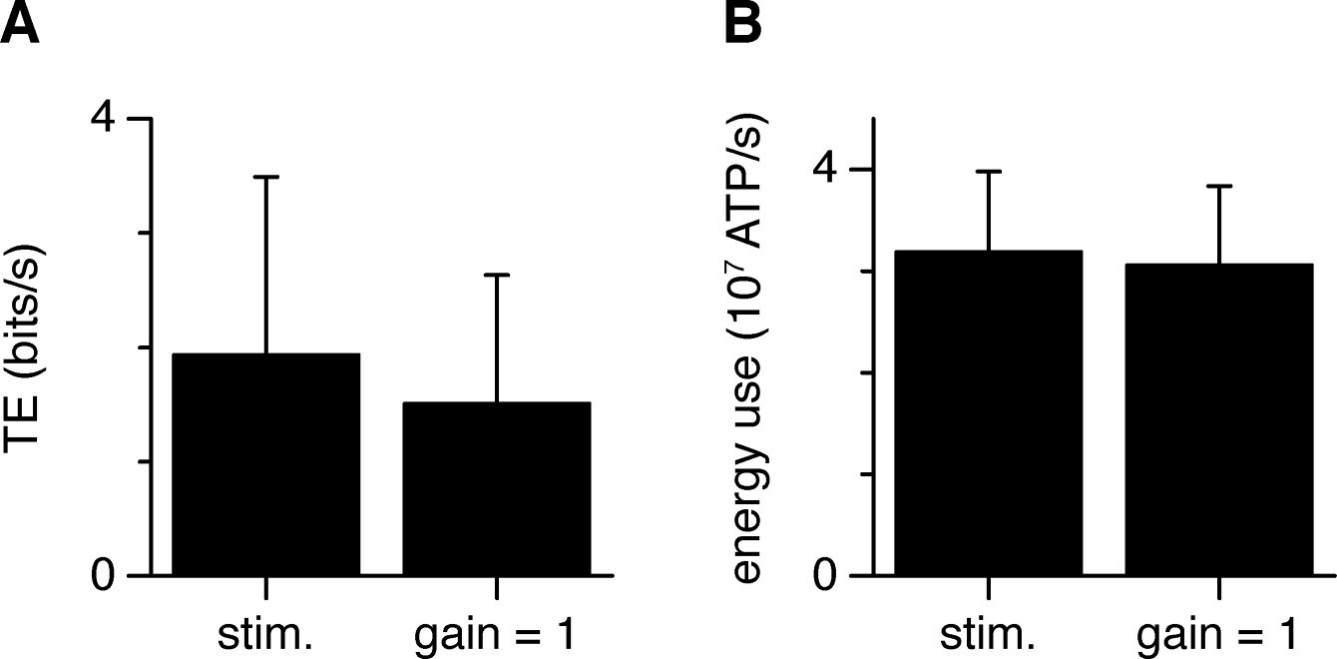
Comparison between real stimulation and dynamic-clamp. (A) Transmitted information when cells (n = 6) were either stimulated through AP-evoked synaptic currents (real stimulation, left), or when the conductance evoked by real stimulation was injected at the soma with dynamic-clamp at gain 1, along with thalamocortical and corticocortical noise (right; difference n.s.; paired Student *t*-test). (B) ATP used on pumping out Na^+^ entering through the postsynaptic conductance, in voltage-clamp in response to thalamic axon stimulation (left), and following injection of the same conductance at the soma using dynamic clamp. When the postsynaptic conductance trace derived from presynaptic stimulation was not scaled (but noise from the corticocortical and thalamocortical inputs was injected), the postsynaptic energy use was very slightly but significantly reduced (by ~4%; p = 0.02; paired Student *t*-test) between the real stimulation (left) and when the conductance evoked by real stimulation was injected at the soma with dynamic-clamp at gain 1 (right).

**Fig 6:**
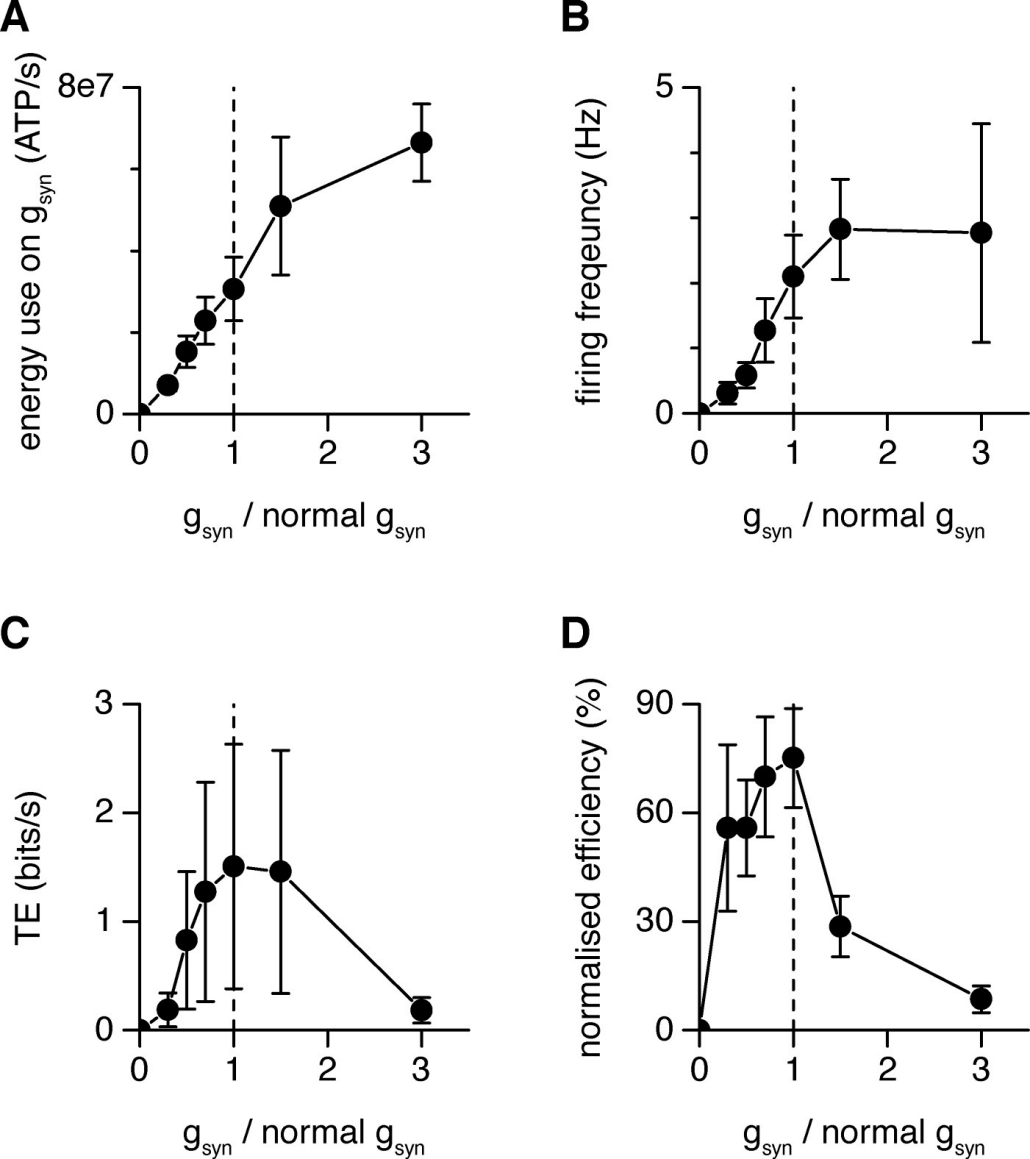
Energy efficiency at thalamocortical synapses. (A) Energy use on ion flux associated with g_syn_ as a function of g_syn_ scaling. (B) L4SS firing frequency as a function of g_syn+tc_ scaling (while g_cc_ injection remains constant). (C) Dependence of information transmission on g_syn+tc_ scaling. Scaling of g_syn+tc_ /(normal g_syn+tc_)_*_1 was applied to all 6 cells, _*_0.3 to 3 cells, _*_0.5 to 4 cells, _*_0.75 to 5 cells, _*_1.5 to 5 cells, and _*_3 to 4 cells. Like for the simulations, *TE* is the result of subtracting *TE*_*noise*_ from *TE*_*raw*_ (see Eq (2) and discussion in Materials & Methods). To ensure significance of the results, we tested that both *TE*_*noise*_ and *TE*_*raw*_ are significantly larger than 0 (Student *t*-test; p_*noise*_ = 1.2_*_10^−13^ and p_*raw*_ = 0.0014) and that *TE*_*raw*_ > *TE*_*noise*_ (paired Student *t*-test; p_diff_ = 0.004). (D) Energy efficiency (information in bits/sec divided by EPSC energy in ATP/sec normalized to the peak for each cell) curves for the averaged data over 6 cells. In all panels, error bars stand for the s.e.m. and broken lines only connect data points for visual guidance.

Data were analysed using custom scripts written in MATLAB (The Mathworks Inc.). Postsynaptic current traces were used to calculate ATP consumption at the synapse as described above. Postsynaptic voltage traces were converted to binarised sequences of 1s (representing action potentials) and 0s (their absence) by identifying events whose amplitude exceeded a threshold defining action potential occurrences (set individually for each cell, between -15 mV and -30 mV). This output sequence could then be compared with the binary input spike train to look at simple transmission characteristics (Fig 4F), or used to calculate the amount of information that would be propagated to the visual cortex by the postsynaptic cell (Figs 5A and 6C).

Spike transmission characteristics were calculated as in [12]. The percentage occurrence of each possible input-output relationship (Fig 4F) was calculated by searching for an output (AP or EPSP) in the 18 ms following an input spike (top row) or for an input (AP) in the 18 ms preceding an output spike (bottom row), and summing each occurrence across all L4SS neurons studied.

Data are presented as mean ± standard error of the mean (s.e.m.), unless mentioned otherwise. Differences between means were assessed with Student’s t-tests (Fig 5). Note that we did not compare the means between different conductance scaling factors for the experimental results in Fig 6; we present ± s.e.m. for each point simply as a visual guide for the reader. This is because we want to be careful not to claim that the peak is precisely at 1. We do not have a large enough dataset nor fine enough sampling around g_syn_ = 1 to claim that this is the exact value of the peak.

## Results

### Modelling thalamocortical synapses

We have previously shown that the strong retinothalamic synapse relaying information from the retina to the thalamus maximises energetic efficiency (information transmitted per ATP used) when transferring information [12]. It is however unclear whether this principle of energy efficiency also applies at synapses that contribute more weakly to determining the output of the postsynaptic cell. To address this question, we adapted a Hodgkin-Huxley-type multicompartment model of a layer 4 spiny stellate (L4SS) cell [15] (see Materials & Methods), the next step in the visual pathway after the thalamus. L4SS cells receive excitatory inputs from thalamocortical relay cells, and excitatory and inhibitory inputs from cortical neurons (Fig 1A). While corticocortical synapse surface density is assumed to be homogeneous throughout the dendritic tree, thalamocortical synapse density follows a Gaussian distribution with respect to the distance from the soma (Fig 1B) [19]. To create background ‘noise’, all thalamocortical synapses were randomly activated at 4 Hz [12], while corticocortical excitatory and inhibitory synapses were randomly activated at 0.45 Hz and 0.09 Hz respectively [18]. Synaptic conductances were initially set to the same value for every synapse in a given category so as to generate on average an ~0.8 mV depolarisation at the soma for thalamocortical synapses, a ~0.6 mV depolarisation for corticocortical synapses and a ~0.1 mV hyperpolarisation for inhibitory synapses (Fig 1C). This constant barrage of excitatory and inhibitory inputs generated random fluctuations of the membrane voltage at the soma and led to irregular spiking (Fig 1D, black trace).

We then modelled one extra thalamocortical axon. This axon of interest contacts the dendritic tree at three independent but relatively close synaptic locations (‘syn’ in Fig 1A). Activation of this single extra axon (at ~4 Hz, the firing frequency of the thalamocortical neurons for physiological input [12]) visibly affects the output of the neuron even in the presence of intense random background activity from all other synapses (Fig 1D, light violet trace; activation times for that specific simulation are labelled with vertical light violet bars, the background synaptic input was identical for the black and light violet traces). Thus, some information about this input spike train is carried across the synapses to be represented in the output spike train of the postsynaptic L4SS neuron. We then evaluated the amount of information transmitted across thalamocortical synapses.

### Measuring transfer entropy at weak synapses in the presence of noise

Information transfer at synapses can be assessed in different ways. One commonly used method consists of computing the *mutual information* between the input and output spike trains across the synapse(s) of interest [20]. This method has been successfully applied in a number of studies (e.g. [21, 22]), including by ourselves [4, 12], and numerous studies have addressed the pitfalls and biases of using mutual information in that context (see [23] for a review).

We have recently used the so-called direct method of Strong *et al*. [21] to measure information transfer (mutual information) at the retinothalamic synapse [12]. That synapse differs significantly, however, from the one we study here, as it is a strong relay synapse, generating EPSCs sufficiently large that a single one of them is often sufficient to trigger an output action potential on its own. Indeed, the output of thalamic relay cells in the visual pathway is driven almost entirely by input from a single strong relay synapse impinging on their dendritic tree from the retina [37]. The case we study here is different as L4SS cells receive weak inputs from hundreds to thousands of thalamic and cortical synapses (Fig 1A and 1C), each one of them generating small EPSCs that individually contribute little to the output of the cell (Fig 1B–1D).

Preliminary simulations revealed that using the direct method would be inappropriate because of the relative weakness of the synapse and the presence of background noise. At the thalamocortical synapse, in the presence of a significant barrage of background inputs, most output spikes are not related to input spikes in the axon of interest (Fig 1D). In these circumstances, we could not feasibly run long enough simulations and experiments to estimate accurately the mutual information between input and output sequences, a recurrent problem in experiments using acute brain slices with a lifespan counted in hours [38]. The direct method we have used in ref. [12] computes the mutual information by subtracting the noise entropy (H_noise_) from the output’s total entropy (H_total_). While H_total_ is estimated by looking at the distribution of binary ‘words’ over time, H_noise_ is computed as the entropy of these words’ distributions across repetitions, and then averaged over time. It is thus crucial for correctly estimating H_noise_ to collect a sufficient number of repetitions. H_total_ and H_noise_ impose competing constraints over the time available for data collection as the best estimate of H_total_ will be obtained with the longest possible non-repeating sequence, while the best estimate of H_noise_ will be obtained with the largest number of repetitions of a repeating sequence. In Harris *et al*. [12], in the case of thalamic relay cells, most output spikes were directly driven by an input spike and the noise was relatively small in comparison to H_total_. It was thus possible to reach a reasonable estimate of the mutual information with few repetitions. In the present case, only a few of the output spikes are related to an input spike from the thalamocortical axon of interest, and one expects that H_noise_ becomes approximately equal to H_total_, necessitating a number of repetitions for proper estimation of the mutual information incompatible with the experiments presented here. Others have faced the same problem before. London and colleagues, for instance, addressed it by devising an alternative measure similar to mutual information called *synaptic information efficacy* [39]. However, another concern is that measures like the mutual information are symmetric, i.e. they measure how much information can be inferred about one process when observing the other one, but do not measure directional information flow.

To deal with these issues, we decided to employ the measure termed *transfer entropy* (see Eq 1 in Materials & Methods) [13]. Transfer entropy is designed to measure directional information transfer from a sender to a receiver. If the information flow is unidirectional (from the sender to the receiver), measuring transfer entropy from the receiver to the sender will return 0. Thus, we binarized the 125 second input and output spike trains in 3 ms time bins and measured transfer entropy from the input axon to the output spike train using the package published by Ito and colleagues (http://code.google.com/p/transfer-entropy-toolbox/) [14]. Due to the limited size of the dataset that can be realistically acquired, we additionally applied a correction for random coincidences that contribute to noise in the estimation of the transfer entropy (see Materials & Methods for a detailed discussion of these issues). In the following, we usually report *TE*_*raw*_, the raw value of transfer entropy calculated from the input to the output using Eq (1) (see Materials & Methods), *TE*_*noise*_, the value of transfer entropy calculated using Eq (1) but using a scrambled input (scrambling the sequence of words instead of the sequence of individual time bins produces almost identical results, see S1 Fig), and *TE* = *TE*_*raw*_−*TE*_*noise*_, the effective information transfer after correcting for the effect of random coincidences (Eq (2) in Materials & Methods).

In multicompartment simulations of the thalamocortical synapse, using *TE* for our axon of interest, we found that a small amount of information is indeed carried across the simulated synapse of interest, from input action potentials, even in the presence of strong background noise generated by other synapses (Fig 1A), yielding a transfer entropy of *TE =* 0.47 ± 0.06 bits/sec (mean ± s.e.m.) after subtraction of *TE*_*noise*_ (*TE*_*raw*_ = 1.0 ± 0.1 bits/sec; mean ± standard deviation), which is roughly 40 times less than at thalamic relay cells [12].

We then investigated whether modulating the strength of thalamocortical connections modulates information transfer, and how this relates to energy consumption by the postsynaptic cell.

### Energetic efficiency at simulated thalamocortical synapses

In order to assess how modulating the strength of thalamocortical connections affects the postsynaptic cell’s energy consumption and information transfer at our axon of interest, we scaled all thalamocortical synapses by a range of gain factors [0, 0.4, 0.6, 0.8, 1, 1.2, 1.4, 1.6, 2.4, 4.8, 7.2, 9.6] while injecting excitatory and inhibitory corticocortical background noise at a constant level.

Increasing the conductance of all the thalamocortical synapses increased both the EPSC size (because more current is injected from the axon of interest) and the energy consumption in the postsynaptic cell associated with the thalamic input (Fig 2A), because more sodium ions need to be actively extruded from the cell via the Na,K-ATPase pump due to extra Na^+^ entry at all the thalamocortical synapses (see [4] for a review of synaptic energetics). This increase in energy consumption is roughly proportional to the scaling factor for the thalamocortical synapse conductance (Fig 2A).

Altering the conductance of the set of thalamocortical inputs to the L4SS cell affects the output firing rate. The rate of information transmission across a single thalamocortical connection increases with the output firing rate as a sigmoid function, up to about 20 Hz, above which the information transmission plateaus (Fig 2B). This is true for the raw value of transfer entropy (*TE*_*raw*_) or after subtraction of *TE*_*noise*_ (*TE*). Increasing the conductance of the set of thalamocortical inputs by 20% increases the amount of information that is transmitted across a single thalamocortical connection by ~16%, showing that thalamocortical inputs to L4SS cells, as with retinothalamic inputs to relay neurons [12], do not maximise information transmission across single connections (Fig 2C). However, dividing the information transmitted by the energy used on this connection demonstrates that the physiological thalamocortical input characteristics are close to optimality for maximising the information transmitted per energy used for each individual connection (Fig 2D). This was true when placing the three synapses of interest at the location depicted in Fig 1A (syn) or at a different location on another dendrite. Specifically, to test for the influence on our results of the location of the three thalamocortical synapses from our single axon of interest (see Fig 1A), we repeated all the simulations with a second set of such synapses (see Materials & Methods for details). These additional simulations revealed no qualitative differences to what had been obtained with the first set. Results for this second set of synapses are plotted in dark violet in Fig 2A–2C, with the average over both datasets plotted in blue. The ratio of information transmitted to energy used is approximately maximised for both datasets when looking at *TE*_*raw*_ (light and dark violet traces), averaging over both sets of locations (blue trace), or when looking at *TE* after noise subtraction and averaging over both datasets (red trace), either when considering the energetic cost associated with the synapses of interest only (Fig 2D), or when considering the total signalling energy budget of the cell (Fig 2E). While small differences are present between the results obtained with the two different synapse placements tested for the investigated thalamocortical axon, the average shows a sharp peak at gain = 1, suggesting that these synapses are, on average, tuned for energetic optimality of information transmission. These results were obtained with words of length 10 time bins (30 ms) and allowing for temporal frame shifts between the input and output sequences of up to 30 ms. Note however that transfer entropy is maximal without a frame shift between the input and output sequences when using a 3 ms temporal resolution and all results reported hereafter are for temporally aligned sequences with no frame shift. Systematic tests showed that changing the value of these parameters, while introducing small quantitative changes to the results, did not lead to significant changes in the conclusions reached above (see Materials & Methods for a detailed discussion of these parameters).

### Inhibition modulates the position of peak energetic efficiency of information transfer

To test for the influence of inhibition at the thalamocortical synapse, we used the mathematical model of L4SS cells and ran a second set of simulations with the same synaptic placements as before but without inhibitory input (setting g_inh_ to 0; see Materials & Methods). The results obtained without inhibition are plotted in Fig 3 and overall, match those from Fig 2 (with inhibition) very closely. As for when inhibition was present, the increase in energy consumption was roughly proportional to the scaling factor of thalamocortical synapse conductance (Fig 3A), the rate of information transmission across a single thalamocortical connection increased with the output firing rate as a sigmoid function (Fig 3B) and thalamocortical inputs to L4SS cells did not maximise information transmission across single connections (Fig 3C), although now increasing the conductance of the set of thalamocortical inputs could only increase the amount of information that is transmitted across a single thalamocortical connection by ~8%.

Interestingly, even though the changes between the results obtained with (Fig 2) and without inhibition (Fig 3) were small, they have an effect on the value of the energetically optimal conductance for information transmission. Specifically, removing inhibition slightly shifted the curve in Fig 3C to the left (towards lower gain factors) when compared to results obtained with inhibition (Fig 2C). As a result, dividing the information transmitted by the energy used by all thalamocortical connections now yielded an optimum synaptic conductance value for energetic efficiency that was slightly lower than obtained previously (Fig 2D). Energetic optimality of information transmission was obtained for *TE* for gain = 0.8 when considering the energetic cost associated with all thalamocortical synapses (Fig 3D) and again for gain = 0.8 when considering the total signalling energy budget of the cell (Fig 3E).

Overall, these results suggest that the finding that experimentally reported synaptic conductances are close to the energetic efficiency for information transmission is robust, even though the exact position of that optimum on the conductance scale can be slightly affected by the exact positioning and clustering of synapses on the dendritic tree, or by the fine balance between excitatory and inhibitory activity levels in the network.

We next explored whether these results could be replicated experimentally.

### Spike transmission through the thalamocortical synapse

In order to experimentally test our simulation results, we stimulated thalamic axons making synapses onto whole-cell patch-clamped L4SS cells of V1 in rat brain slices (Fig 4A and 4B). As for the retinothalamic synapse [12], we used P28 animals, minimal stimulation to activate a single input, and gabazine to block GABA_A_ receptors. The stimulus size in the experiments was adjusted to produce the smallest detectable EPSC (mean value 356±186 pA). Single EPSCs were smaller (Fig 4C) and showed less paired pulse depression (Fig 4D and 4E) than at the retinothalamic synapse (compare with S1 Fig in ref. [12]).

The L4SS cells were held at -70 mV, and the input synapse stimulus trains used were thalamic relay neuron responses to retinal input obtained by [12], which had a mean spike frequency of ~4.3 Hz. Upon presynaptic stimulation, fewer spikes were successfully transmitted and more spontaneous spikes occurred at the thalamocortical synapse (9% and 68%, respectively, Fig 4F) than at the retinothalamic synapse described previously (19% and 7% respectively, [12]), confirming that one lateral geniculate nucleus (LGN) relay neuron has a weaker effect on L4SS cell firing than does one retinal ganglion cell on LGN relay neuron firing.

### Relationship between synaptic conductance and spike output

In contrast to the strong one-to-one relationship between retinal ganglion cells (RGCs) and thalamic relay neurons, where RGC activation is sufficient to evoke a relay neuron spike 19% of the time [12], L4SS cells receive weaker input from ~100–600 spines targeted by thalamic neurons (cat V1: Peters and Payne [40]; rat barrel cortex: Bruno and Sakmann [41]), that individually evoke 0.5~2 mV EPSPs (cat V1: Stratford et al. [33]; rat barrel cortex: Bruno and Sakmann [41]). These EPSPs are sufficient to evoke a spike only 9% of the time (Fig 4F). The receptive field of a L4SS cell differs from those of its input relay neurons (cat V1: Hubel and Wiesel [42, 43]), and L4SS cell spiking is also generated by intracortical inputs (mouse V1: Lien and Scanziani [44]) with input strengths that tend to be slightly smaller (~1 mV in L4 and ~ 0.22 mV in L6), but more numerous, than the thalamocortical inputs (cat V1: Stratford et al. [33]; Tarczy-Hornoch et al. [45]). Is the ratio of information transmitted to energy used maximised at a single thalamocortical synapse, operating in the context of hundreds of similarly weighted synapses?

We recorded the sequence of L4SS cell EPSCs evoked by minimal stimulation with a physiological firing pattern (i.e. the thalamic relay neuron responses to retinal input described above), and converted this to a synaptic conductance train (g_syn_; Fig 4G). To simulate the action of this synapse with background synaptic activity present, we used our computational model to synthetically generate two excitatory “noise” conductance trains (see above and Materials & Methods). One train represented other thalamocortical input synapses, which make up ~20% (5% in cat V1: Peters and Payne [40]; 30% in mouse V1: Lien and Scanziani [44]) of the excitatory synapses to L4SS cells (g_tc_), while the other represented corticocortical inputs, which make up the remaining 80% of excitatory synapses to L4SS cells (g_cc_) (Fig 4G). (L4SS neurons also receive inhibitory input *in vivo*, which was not injected experimentally, but was simulated in our Hodgkin-Huxley-type multicompartment model, see above.) The baseline levels of the two excitatory noise traces were scaled together for each cell (mean scaling factor 0.4 ± 0.1 in 6 cells) so that, when injected simultaneously using dynamic-clamp, the L4SS cell firing frequency was approximately 4 Hz, the mean cortical firing rate *in vivo* [1]. Then, g_syn_ was added to the noise trains and the resulting composite conductance time course was injected into the L4SS cell (using dynamic-clamp to generate the appropriate membrane current from the conductance time course), while the voltage response was recorded (Fig 4H). Adding g_cc_+g_tc_ led to an increase in the proportion of spikes transmitted from the stimulated thalamocortical axon (from 9% to 16% of input spikes), presumably because depolarisation by the synaptic noise conductances (which would be occurring *in vivo*) enabled more synaptic depolarisations to reach threshold (Fig 4F).

### Information transmission

The input action potential train was derived from the output spike train recorded in thalamic relay cells, which had a frequency of 4.3 Hz, and had an entropy rate of about 32 bits/sec [12]. The output spike train recorded in cortical L4SS neurons (without the simulated synaptic noise added) had a frequency of 1.2 ± 0.8 Hz (mean ± s.e.m.) and a transfer entropy rate of 1.9 bits/sec (1.9 ± 1.6 bits/sec; mean ± s.e.m.; Fig 5A) encoding ~1.6 bits/spike (1.9 bits/sec at 1.2 Hz).

Employing dynamic clamp to apply the recorded synaptic conductance at the cell soma, in addition to the thalamocortical and cortical noise conductances, gave a transfer entropy rate of 1.5 ± 1.1 bits/sec, which was not significantly different from that seen with real presynaptic stimulation (paired Student *t*-test, p = 0.41; Fig 5A). These rates were slightly higher than the rates observed in simulations (*TE* = 0.47 ± 0.06 bits/sec), probably due to the fact that unlike in simulations, in experiments, all input is injected at the soma.

### Energy use on postsynaptic current

We calculated the energy used on a single thalamocortical connection–either evoked by presynaptic stimulation in voltage-clamp mode or scaled and injected using dynamic clamp in current-clamp mode–by calculating the total number of Na^+^ ions that must be actively extruded at the expense of 1 ATP molecule for every three Na^+^ ions [1, 4]. When the postsynaptic conductance trace derived from presynaptic stimulation was not scaled (but noise from the corticocortical and thalamocortical inputs was injected), the postsynaptic energy use was very slightly reduced by ~4% with respect to that occurring with real presynaptic stimulation in voltage-clamp mode (p = 0.02; Fig 5B). This is because, in current-clamp mode, depolarisation of the cell by the injected conductances and any action potentials that are evoked reduce slightly the Na^+^ entry through postsynaptic channels compared to the stimulation condition in which the cell is voltage clamped at its resting potential.

### Information transmission and energy efficiency at different synaptic conductances

We next used dynamic clamp to investigate experimentally the effects on information transmission and energy use of scaling the thalamic input conductances up or down, while keeping the corticocortical input conductance constant. Since we are seeking the optimal conductance value for all thalamocortical synapses, the values of g_syn_ and g_tc_ were scaled together. This results in the thalamic noise level increasing when g_syn_ is increased.

As observed with the simulations (Figs 2A and 3A), the energy use calculated to be associated with g_syn_ increased approximately linearly with the effective postsynaptic conductance but appears to tail off as the gain increases above 2 (Fig 6A). The L4SS cell firing frequency increased with thalamocortical conductance (with g_syn_ and g_tc_ scaled up or down together as described for Fig 4G), although, like for thalamic relay cells [12], this relationship begins to plateau at conductance gain values higher than 1 (Fig 6B).

Examining transfer entropy as a function of thalamocortical conductance showed that, on average, the physiological gain of the synapse is at, or very close to, maximizing information transmission from one LGN relay neuron to the cortex (Fig 6C), unlike in simulations where a modest increase in information transmission was observed when increasing the physiological level of the thalamocortical conductance (Figs 2C and 3C). This discrepancy is probably explained by the fact that, in experiments, the whole synaptic conductance had to be injected via the patch-pipette at the soma. We then assessed the relationship between information transmission and its associated energetic costs, as previously done for the thalamic relay synapse [12] and in simulations (Figs 2D, 2E, 3D and 3E).

We calculated the ratio of the information transmitted through one thalamocortical synapse to the energy used on its postsynaptic current, when dynamic clamp was employed to inject a thalamocortical conductance of different magnitudes (Fig 6D). Like for multicompartment simulations (Figs 2D, 2E, 3D and 3E), the resulting average peak energy efficiency for information transfer occurred at a conductance value close to the physiological value for the synapse. We cannot claim that the peak is precisely at the physiological value: this would require finer sampling of the conductance values around g_syn_ = 1, and a larger experimental dataset to justify statistical comparison between conductance gain factors. We simply wish to show that the shape of the experimental curve is comparable to the simulated results, recapitulating and supporting the predictions made by the simulations in Figs 2 and 3. Together, the simulation and experimental results suggest that, as for the much stronger retina-LGN synapse, the weak thalamocortical synapse displays an optimal value of postsynaptic conductance for maximizing the information transmitted per energy used on synaptic currents which is in the region of the physiologically observed value.

## Discussion

We have previously shown that information transmission is sacrificed in favour of energetic efficiency at the retinothalamic synapse between retinal ganglion cells and relay neurons in the dorsal LGN of the thalamus [12]. We wondered whether such energy efficiency is a special property of strong relay synapses like that in the LGN, where postsynaptic spiking is largely driven by a single input, or whether similar principles apply at synapses that contribute less dominantly to postsynaptic spiking. At the next synapse along the visual pathway, the thalamocortical synapse, the set of thalamic relay neurons makes up to 600 contacts with the spines of a single cortical L4 spiny stellate cell, the firing of which is also influenced by abundant cortical input. Strikingly, both simulations and experiments presented here show that, even in the presence of random background corticocortical and thalamocortical input, the ratio of information transmitted to energy used is maximised near the physiological conductance of the weak thalamocortical synapse.

Using an updated multicompartment Hodgkin-Huxley-type model of L4SS cells [15], we simulated a constant background level of excitatory and inhibitory cortical inputs, while varying the conductance strength of a set of thalamocortical inputs. We found that the information transmitted from one thalamic input was maximised when the conductance of the set of thalamic inputs was slightly larger than the physiological value (Figs 2C and 3C). However, when the information transmitted across a single thalamic input was divided by its associated postsynaptic energy use, we found a sharp peak in energy efficiency of information transmission, which was at its maximum when the set of thalamic inputs were close to their physiologically observed conductance value (Figs 2D, 2E, 3D and 3E). Thus, thalamocortical synaptic properties appear to be set to favour energy efficiency for information transmission.

To check these results experimentally, we recorded the postsynaptic current evoked in single L4SS cells in response to stimulation of a presynaptic thalamic axon. We converted this current to a conductance, which could then be scaled up or down (mimicking larger or smaller synapses) and injected into the soma of the cell using dynamic clamp. Extra thalamic and cortical conductances derived from the simulations were simultaneously injected into the L4SS cell. In the presence of this constant background level of cortical inputs, we found that the information transmitted from one thalamic neuron already is maximal in the range of the physiological gain of the synapse–increasing or decreasing the gain by the factors that we applied reduced the information transmitted (Fig 6C). Nevertheless, if information transmitted is divided by the energy used on postsynaptic ion pumping [4, 12], we found that this measure of synaptic energetic efficiency also decreased when larger or smaller experimental gain factors were applied (Fig 6D). The exact position of the peak cannot be revealed by our experimental data but, taken together with the predictions from our multicompartment simulations, it appears that–as for the retinothalamic synapse–weak thalamocortical synapses display maximal information transmitted per energy used at or near physiological conductance values.

### Effects of local inhibition at the thalamocortical synapse

To test for the influence of inhibition at the thalamocortical synapse, we used the mathematical model of L4SS cells and ran simulations with and without inhibitory input (see Materials & Methods). Results obtained in the Hodgkin-Huxley-type multicompartment model in the presence of inhibition recapitulate the experimental results of Fig 6C and 6D. Even though information transfer increases monotonically in the simulations (Figs 2C and 3C) while it eventually decreases when the gain is increased in experiments (Fig 6C), this did not affect the excitatory conductance magnitude which produced the peak metabolic efficiency. Removing the inhibitory input in simulations did however change slightly the synaptic conductance value for the peak metabolic efficiency, shifting it to lower values (Fig 3D and 3E), suggesting that the balance between excitation and inhibition in the network may play a role in fine-tuning synaptic conductance values for energetic optimality of information transfer. Specifically, we reason that, because increased inhibition would demand that more excitatory charge enter the postsynaptic cell to achieve the same voltage change, when inhibition does not have to be overcome, the peak position of energy efficiency shifts to a lower excitatory conductance value. The discrepancy observed between simulations (Fig 2C) and experiments (Fig 6C) in the relation between information transfer and gain, at gains higher than ~1.5, can probably be attributed to the slightly different stimulation scenarios, as in experiments we were constrained to inject all input at the cell soma.

### Correlations between inputs

*In vivo*, especially in sensory systems, it is likely that a postsynaptic neuron will receive inputs from cells that are transmitting correlated information. For instance, the receptive fields of “simple cells” in the visual cortex are thought be built from the receptive fields of spatially adjacent thalamic relay neurons [42, 43], and therefore the excitatory thalamic input to a cortical cell may be correlated not just in amplitude (as we have mimicked here), but also in time, as a single object passes through the visual field. Additionally, inhibitory input may be correlated with excitatory input. In assessing how synaptic conductances are set to regulate information transfer and energy use, it will be interesting to investigate whether the conductance of each input to a postsynaptic cell is set independently, or whether account is taken of the correlations in information passing through spatially adjacent cells.

Temporal correlations are also seen in the form of oscillatory activity throughout the thalamocortical loop in different states of arousal [46]. This activity may be important for putting the visual scene into context, for attention, or even for conscious “binding” of different attributes of a visual scene [47]. In the present paper, we have focused on the simple feed-forward connection from thalamus to cortex, but of course a feed-forward input could arrive at any stage of such temporal oscillation, thus impinging on a more or less hyperpolarized cortical neuron. It would be interesting to investigate how such network-level membrane potential rhythms affect the energy efficiency of input connections. However, we do not find it necessary to postulate that the same energetic principles should hold for all oscillation conditions. For instance, it is possible that the high amplitude, low frequency thalamocortical oscillations that are seen in deep sleep may play a role in synaptic renormalization, perhaps actively regulating synaptic strength with energy efficiency a prioritized parameter. Note that detailed investigation of these questions in the future might require the use of conditional transfer entropy.

### Additional pressures on neuronal communication

How can the preference of single synapses for an optimal information-to-energy ratio be reconciled with the need for strongly varying synaptic strengths for learning? This is a question that we are very intrigued by, and our best explanation is that synapses are efficient *on average*. If each synapse were always set to its energetic optimum, then how could the relative weights of synapses store information? From what is known about experience-driven and homeostatic mechanisms of synaptic plasticity, it is clear that we cannot consider energy to be the only determinant of neuronal organisation. Nor is information the only alternative competing pressure. For instance, brain-wide activity as a whole must be protected from entering dangerous regimes of recurrent excitation, and such stable network dynamics have recently been shown to be prioritised above optimal information transmission [48]. Many organisational constraints need to be considered to gain a full understanding of the evolutionary pressures that have guided brain design. We do not claim to have considered all such constraints here, but simply argue that energetic efficiency appears to be one important pressure on the strength of any synapse.

Both our previous work (retinogeniculate synapse [12]) and the present study (thalamocortical synapse), focus on relatively low-level feed-forward synapses in the adult visual system, examined after the critical period of development. We would predict that the distribution of synaptic strengths would be broader in a highly plastic brain region, or during development, with fewer synapses sitting at the energetically optimal point at any one time.

### Design or coincidence?

We have found that postsynaptic properties maximise the information transmitted per energy used. While these properties are of course the result of evolutionary selection, how do we know that energy efficiency was the selective pressure for these properties? That the evolved synapse properties facilitate energetically efficient information transfer could be a happy coincidence. One way to investigate this question would be examine the energy efficiency of synaptic communication in phylogenetically related species, to see if efficiency improves on an evolutionary time scale.

Looking beyond synapses in the mammalian brain, there is evidence that many levels of neural organisation adhere to a principle of minimising energy use. At the most macroscopic level, the organisation of brain area localisation has been shaped by energy constraints by minimising the length of “wiring” required for signals to travel from one neuron to another [49]. The segregation of the brain into grey and white matter areas has also been suggested to promote energy efficiency [50]. At the single-cell level, surprising parameters such as the sparse firing [10] and low release probability [4] of many cortical neurons can also be explained when energy is viewed as a limiting factor. Even the dimensions of synaptic boutons and axons and the timescale of neuronal computation have apparently been shaped by the pressure to transmit information reliably given a limited energy supply [51, 52]. It therefore does not seem surprising that the postsynaptic site, where most energy in the brain is used [1, 2], would also show evidence of evolving within strict energetic constraints.

In conclusion, we have shown that weak, as well as strong, synapses in the visual system operate in the region of optimal information transmitted per energy used. This is consistent with energy use being a major constraint on the evolution of the CNS [53–55].

## Supporting information

S1 Fig Alternative for the calculation of *TE*_*noise*_. Same dataset and analysis as in Fig 2, except that *TE*_*noise*_ is calculated by scrambling the sequence of words instead of the sequence of individual time bins. (A) Dependence of transfer entropy (between the specific input considered and the L4SS cell output) on mean output firing frequency evoked by input trains with different g_syn+tc_ multiplier values. Note that calculating *TE*_*noise*_ by scrambling the sequence of words does not significantly change the results obtained (compare with Fig 2). In particular, the position of the peaks does not shift (B-C). However, we note that *TE*_*noise*_ calculated in this manner is larger at high frequencies. While this does not change the shape of the information over energy curves (B-C), it makes the peaks sharper as *TE = TE*_*raw*_—*TE*_*noise*_ drop faster at high gains. See legend of Fig 2 for further details.
(TIF)

S2 Fig Dependence of *TE*_*raw*_ and *TE*_*noise*_ on frame shifts between input and output sequences. Same dataset as in Fig 2. Sample traces of *TE*_*raw*_ (black; mean ± s.d.) and *TE*_*noise*_ are plotted for various gains: gain = 0.6 (A), gain = 1 (B) and gain = 2.4 (C).
(TIF)

S3 Fig Dependence of *TE*_*raw*_ and *TE*_*noise*_ on binarization. Same dataset and analysis as in Fig 2 but with time bins shorter than 3 ms. (A-C) time bin = 0.25 ms. (D-F) time bin = 0.50 ms.
(TIF)
