# Supplementary figures and images for: Energy-efficient information transfer at thalamocortical synapses
Source: PLoS Comput Biol. 2019 Aug 5;15(8):e1007226. doi: 10.1371/journal.pcbi.1007226 (PMC6695202; doi:10.1371/journal.pcbi.1007226)

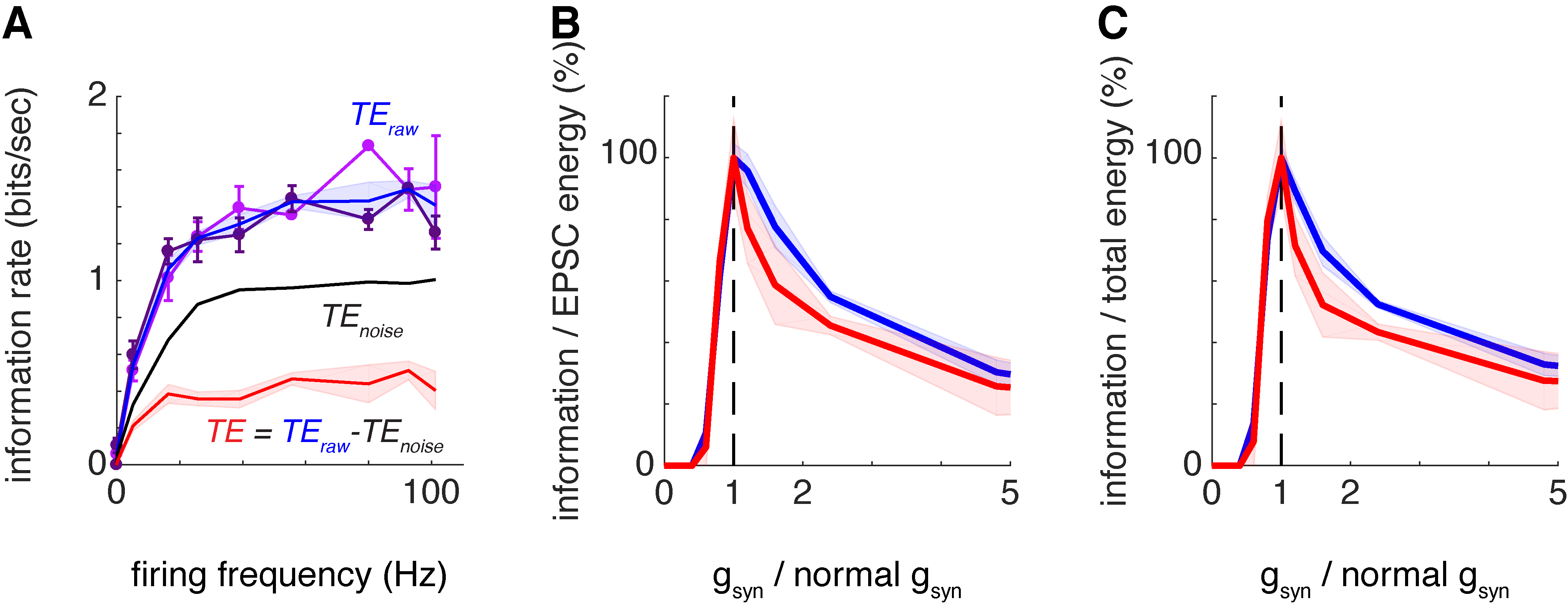

Supplement: S1 Fig — Same dataset and analysis as in Fig 2, except that TEnoise is calculated by scrambling the sequence of words instead of the sequence of individual time bins. (A) Dependence of transfer entropy (between the specific input considered and the L4SS cell output) on mean output firing frequency evoked by input trains with different gsyn+tc multiplier values. Note that calculating TEnoise by scrambling the sequence of words does not significantly change the results obtained (compare with Fig 2). In particular, the position of the peaks does not shift (B-C). However, we note that TEnoise calculated in this manner is larger at high frequencies. While this does not change the shape of the information over energy curves (B-C), it makes the peaks sharper as TE = TEraw—TEnoise drop faster at high gains. See legend of Fig 2 for further details. (TIF) [file pcbi.1007226.s001.tif]

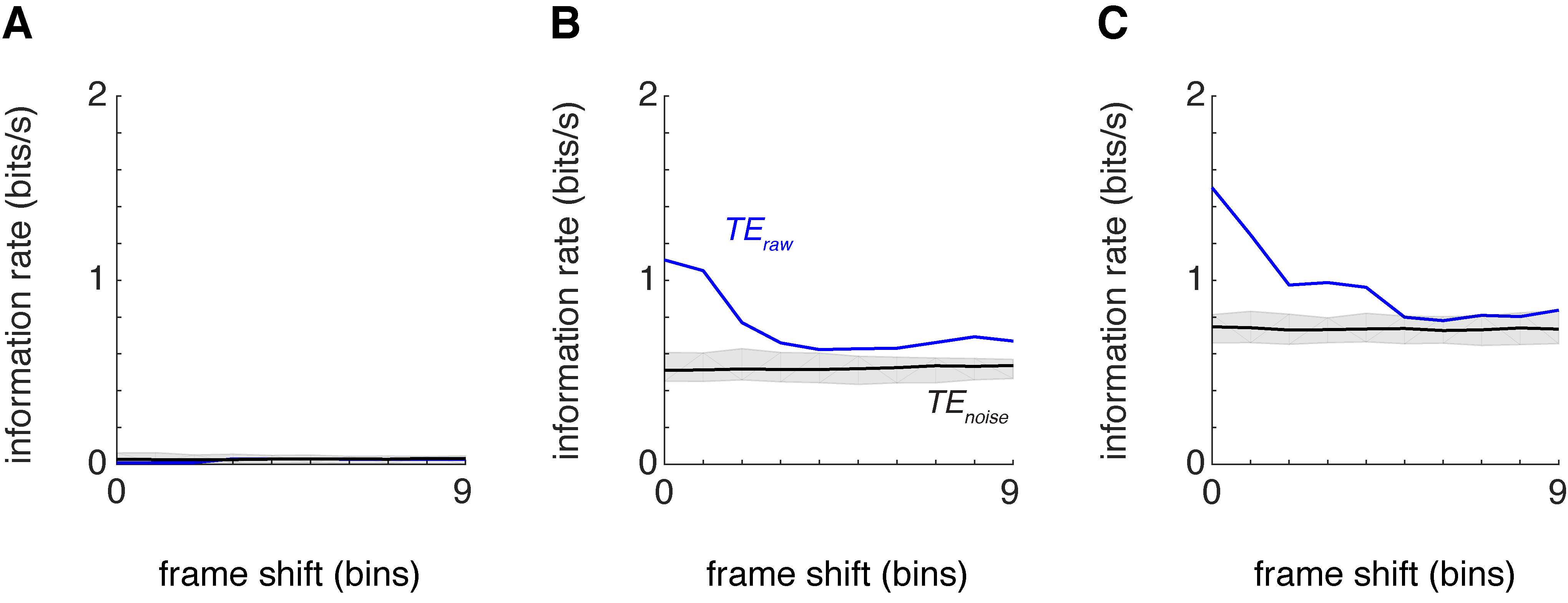

Supplement: S2 Fig — Same dataset as in Fig 2. Sample traces of TEraw (black; mean ± s.d.) and TEnoise are plotted for various gains: gain = 0.6 (A), gain = 1 (B) and gain = 2.4 (C). (TIF) [file pcbi.1007226.s002.tif]

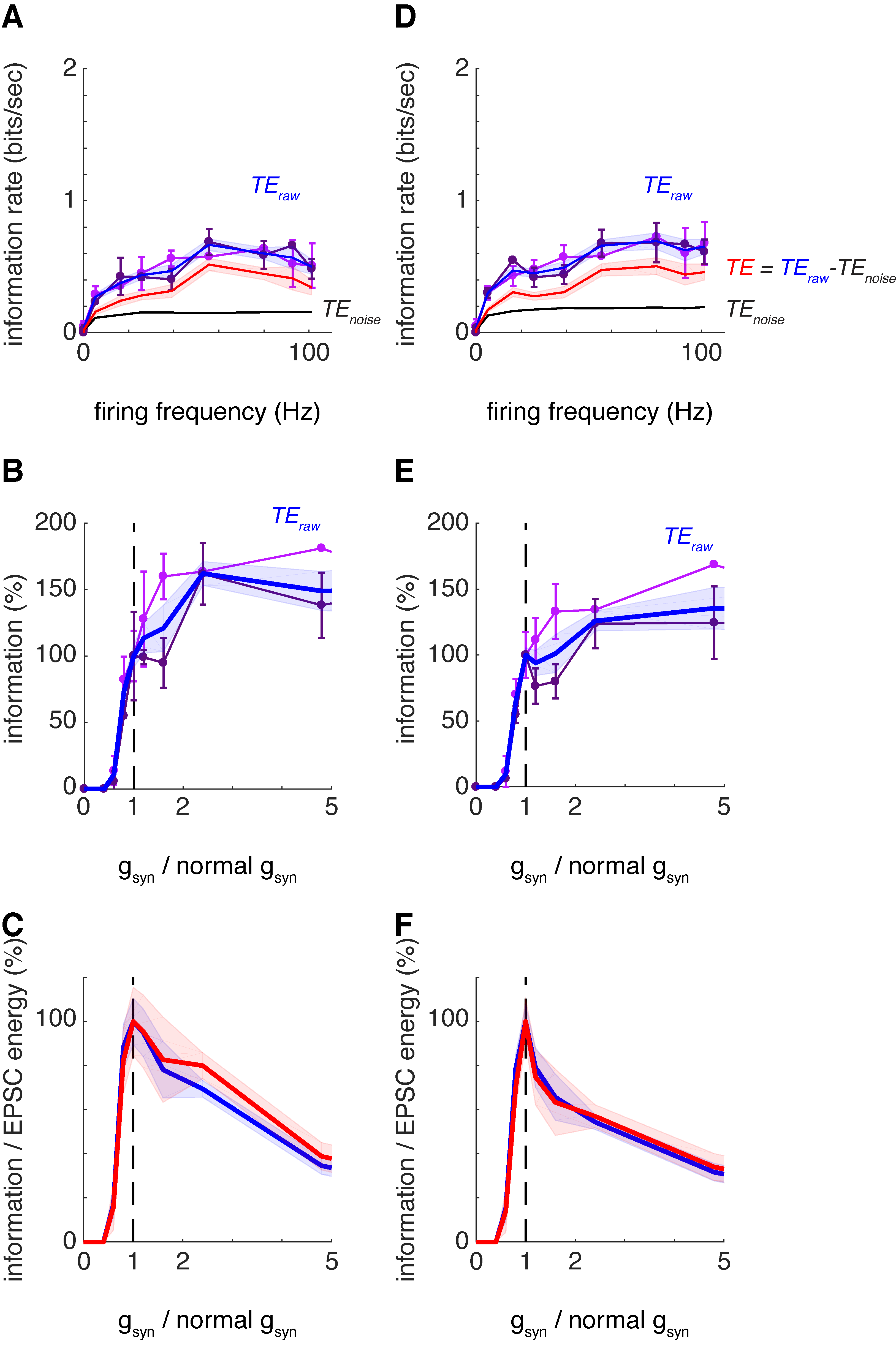

Supplement: S3 Fig — Same dataset and analysis as in Fig 2 but with time bins shorter than 3 ms. (A-C) time bin = 0.25 ms. (D-F) time bin = 0.50 ms. (TIF) [file pcbi.1007226.s003.tif]
